# A randomised controlled trial of a cognitive behavioural intervention for women who have menopausal symptoms following breast cancer treatment (MENOS 1): Trial protocol

**DOI:** 10.1186/1471-2407-11-44

**Published:** 2011-01-31

**Authors:** Eleanor Mann, Melanie Smith, Jennifer Hellier, Myra S Hunter

**Affiliations:** 1 Department of Psychology (at Guy's), Institute of Psychiatry, King's College London, 5th Floor Bermondsey Wing, Guy's Campus, London, SE1 9RT, UK; 2 Department of Biostatistics, Institute of Psychiatry, Kings College London, Box P064, De Crespigny Park, London, SE5 8AF, UK

## Abstract

**Background:**

This trial aims to evaluate the effectiveness of a group cognitive behavioural intervention to alleviate menopausal symptoms (hot flushes and night sweats) in women who have had breast cancer treatment. Hot flushes and night sweats are highly prevalent but challenging to treat in this population. Cognitive behaviour therapy has been found to reduce these symptoms in well women and results of an exploratory trial suggest that it might be effective for breast cancer patients. Two hypotheses are tested:

Compared to usual care, group cognitive behavioural therapy will:

1. Significantly reduce the problem rating and frequency of hot flushes and nights sweats after six weeks of treatment and at six months post-randomisation.

2. Improve mood and quality of life after six weeks of treatment and at six months post-randomisation.

**Methods/Design:**

Ninety-six women who have completed their main treatment for breast cancer and who have been experiencing problematic hot flushes and night sweats for over two months are recruited into the trial from oncology and breast clinics in South East London. They are randomised to either six weekly group cognitive behavioural therapy (Group CBT) sessions or to usual care. Group CBT includes information and discussion about hot flushes and night sweats in the context of breast cancer, monitoring and modifying precipitants, relaxation and paced respiration, stress management, cognitive therapy for unhelpful thoughts and beliefs, managing sleep and night sweats and maintaining changes.

Prior to randomisation women attend a clinical interview, undergo 24-hour sternal skin conductance monitoring, and complete questionnaire measures of hot flushes and night sweats, mood, quality of life, hot flush beliefs and behaviours, optimism and somatic amplification. Post-treatment measures (sternal skin conductance and questionnaires) are collected six to eight weeks later and follow-up measures (questionnaires and a use of medical services measure) at six months post-randomisation.

**Discussion:**

MENOS 1 is the first randomised controlled trial of cognitive behavioural therapy for hot flushes and night sweats that measures both self-reported and physiologically indexed symptoms. The results will inform future clinical practice by developing an evidence-based, non-medical treatment, which can be delivered by trained health professionals.

**Trial Registration:**

Current Controlled Trials ISRCTN13771934

## Background

Hot flushes and night sweats (HF/NS) affect 65-85% of breast cancer survivors, with 60% rating them as severe [1]. They are associated with sleep problems, reduced health-related quality of life [2-4] and are more chronic in this population [5]. Chemotherapy and endocrine treatments such as tamoxifen can induce or exacerbate menopausal symptoms and those taking hormone therapy (HT) are generally advised to stop treatment. HT is an effective treatment, but there is uncertainty associated with its safety. Results of prospective trials [6,7] highlight the association between HT and breast cancer and cardiovascular risks. Therefore a clear need exists for safe and effective non-hormonal targeted therapies that are well tolerated [8].

The exact aetiology of HF/NS is unknown, but they appear to be associated with the rate of change of plasma oestrogen, which influences the thermoregulatory system via the hypothalamus [9]. Alterations in oestrogen levels and neurotransmitters (norepinephrine and serotonin) have been implicated in the pathogenesis of HF/NS [5]. Freedman [10] proposed that there is a narrowed thermoneutral zone (temperature range in which thermoregulation is not triggered) in women who have HF/NS resulting in flushes being triggered by small elevations in core body temperature, caused by changes in ambient temperature or triggers, such as anxiety or stimulants. There is some evidence that the thermoneutral zone is narrowed by elevated brain norepinephrine [10,11], and that stressors increase incidence of hot flushes [12]. Anxiety [13] and cognitions (negative thoughts associated with embarrassment, social anxiety, feeling out of control and unable to cope) are associated with reports of more frequent and problematic HF/NS [14,15], and lifestyle, mood and cognitive and behavioural reactions are likely to influence perception of symptoms [16]. Although the aetiology of hot flushes and night sweats is likely to be the same, their impacts on women are very different. Hot flushes during the day tend to be associated with problems of social anxiety, discomfort and managing day to day activities, whereas night sweats occurring during the night tend to be associated with sleep disruption and associated problems.

Evaluating treatment efficacy depends on valid and reliable measures of HF/NS. The most commonly used measures of HF/NS are women's own self-reports. These include diaries, in which women note the time when a hot flush occurs and usually a severity rating (e.g. mild, moderate or severe). Electronic event markers can also provide a convenient method of registering a perceived hot flush. Sternal skin conductance (SSC) is considered to be the most valid physiological index of HF/NS [17]. A hot flush is marked by a sudden increase in skin conductivity, due to a sweat response, which tails off as sweating decreases (a 'swishy tail'). This pattern is in contrast with a 'saw-tooth' fluctuation in sweating which occurs in typical thermoregulatory control. SSC does not, however, correlate well with subjective measures of severity or problem ratings, and hot flushes defined by SSC and self-reported hot flushes are not always concordant [18]. Reasons for discordance are not well understood, but use of both measures can increase understanding of the impacts of interventions [16]. For example, self-report but not physiological measures (SSC) have shown placebo effects [19], but physiological measures are less predictive of problem ratings and help seeking than self-reports. For these reasons SSC should be used together with self-report measures (frequency and the extent to which they are problematic) [20]. SSC has not yet been used to evaluate treatments in the UK. 24 hour SSC monitoring has been piloted and has been acceptable to women [21,22].

### Non-hormonal treatments for menopausal symptoms in breast cancer patients

Non-hormonal treatments for HF/NS tend to be preferred but the efficacy and acceptability of treatments limit their use. Following a systematic review of non-hormonal medicines for HF/NS, Nelson, Vesco, Haney and others [23] concluded that SSRIs or SNRIs, clonidine and gabapentin trials provide evidence for efficacy but that adverse effects might restrict use for some women. Carpenter, Storniolo, Johns et al [19] reported modest reductions (22%) in HF/NS with Venlafaxine (37.5 mgs) but most patients discontinued treatment at 12 months follow up. These conclusions support those of a position statement by the North American Menopause Society [24]. Furthermore, possible negative interactions between some SSRIs and the effects of tamoxifen may limit prescribing options for women who have had breast cancer [25].

Non-medical treatments are popular, including acupuncture, yoga, exercise, and cognitive behavioural therapy. Studies of acupuncture often report significant reductions in HF/NS following treatment, although few studies report between group effects when compared to sham, superficial or placebo acupuncture [26]. A recent meta-analysis [27] of 4 RCTs of acupuncture for HF/NS in women who have had breast cancer found one RCT that reported significantly fewer HF/NS following acupuncture compared to sham acupuncture [28]. In well women one study out of six reviewed [29] reported a reduction in severity following acupuncture compared to placebo [30]. Both studies involved a comparatively large number of acupuncture sessions, suggesting that it may be effective over and above placebo effects if delivered intensively. Furthermore it would also be worth investigating the reasons for these consistent placebo improvements, and whether they are enduring.

Regular exercise has been associated with fewer HF/NS in cross-sectional studies, but interventions to reduce symptoms using exercise have been less successful [31]. In a systematic review of seven studies, about half reported significant within-group improvements following exercise, but only two reported improvements in HF/NS compared to a no treatment control (one was not powered to find a significant difference [32]). Although there were no studies focusing on breast cancer patients, an RCT of exercise for breast cancer patients with treatment induced HF/NS is due to publish findings in 2011 [33]. Theoretically, yoga may be more effective than exercise; as well as being low intensity exercise, yoga is used as a relaxation technique, which may also reduce HF/NS [34]. A systematic review revealed a reduction in reported HF/NS in 4 out of 7 studies [35], however most RCTs did not find an effect. The exception was an RCT of an intensive yoga programme (60 minutes a day, 5 days a week for 8 weeks) [36]. The only study to measure frequency of physiological HF/NS as well as self-reported HF/NS found no reduction in sternal skin conductance or diary reported hot flushes and night sweats following yoga [37]. There is growing evidence that relaxation therapy and paced respiration can alleviate HF/NS in well women [38-41]. In two preliminary studies with breast cancer patients relaxation significantly reduced HF/NS after 12 sessions [42] but not after one session [43]. A treatment package including behavioural (relaxation and counselling) and pharmacological interventions for breast cancer patients has been evaluated in the US with positive results [44].

A CBT intervention for HF/NS, which includes relaxation and paced respiration, has been developed in the UK by Hunter and colleagues [45-47]. This four-session CBT intervention was shown to be effective in an exploratory patient preference trial of well women (around 40% reduction of HF frequency and problem rating) [45]; a finding replicated by Keefer and Blanchard [48] using 8 sessions of CBT. Women who have had breast cancer tend to experience more frequent and problematic menopausal symptoms than well women going through the natural menopause [3]. In a study of 113 women, who had completed active treatment for breast cancer within the past 5 years, 80% reported HF and 72% NS, a significantly higher prevalence than found in well postmenopausal women [49]. Treatment preferences were explored and preferences were expressed for CBT (63%), complementary therapies (46%), antidepressant treatment (25%) and HT (26%). 53% expressed interest in group and/or individual CBT sessions [3].

Based on earlier work, an exploratory trial of a six-session Group CBT intervention was evaluated in a pre-post study of 24 breast cancer patients [47]. The weekly problem rating reduced on average from 5.9 (SD = 2.1) to 3.4 (SD = 1.8) at post-treatment and 2.8 (SD = 1.8) at follow-up (10 point scale); frequency of HF/NS per week reduced from 68.2 (SD = 28.7) to 46.7 (SD = 30.2) at post-treatment and to 36.9 (SD = 32.5) at 3 months follow up; a significant reduction in HF problem rating and frequency. Feedback from the women, elicited during and at the end of the groups, was very positive [47]. Relaxation/breathing and cognitive strategies used at the onset of HF/NS, as well as group support to make behavioural changes, were seen as most useful. The majority (88%) completed the 3 month follow-up assessment, suggesting good adherence to the study. These results suggest that CBT may be effective in reducing HF/NS and their impacts in breast cancer patients. However, a randomised controlled trial is needed. Furthermore, most studies of the efficacy of CBT used self-reported improvements in HF/NS, thus investigation into the impacts on physiological symptoms is warranted.

#### Current study

This present study aims to evaluate the effectiveness of Group CBT to alleviate HF/NS in women who have had treatment for breast cancer, comparing Group CBT and usual care in a randomised controlled trial, with both physiological and self-reported measures of HF/NS and a 6-month post-randomisation follow-up. We hypothesize that Group CBT will be more effective than usual care in reducing HF/NS problem rating and frequency. Secondary analyses will examine the effects of Group CBT on hypothesised mediating variables, including mood, quality of life, beliefs and behaviours, optimism and somatisation. If effective, the treatment can be promoted by publication of the treatment manual and by training and supervising health professionals in the application of the treatment.

The trial tests four main hypotheses:

1. There will be a significant reduction in problem rating of HF/NS following Group CBT compared to usual care.

2. There will be a significant reduction in frequency of self-reported and SSC-defined HF/NS following Group CBT compared to usual care.

3. Changes in problem rating and frequency of HF/NS following Group CBT will be maintained at six months post-randomisation.

4. There will be significant improvements in mood, beliefs and behaviours about HF/NS and quality of life following Group CBT compared to usual care.

## Methods/Design

**Figure 1:**
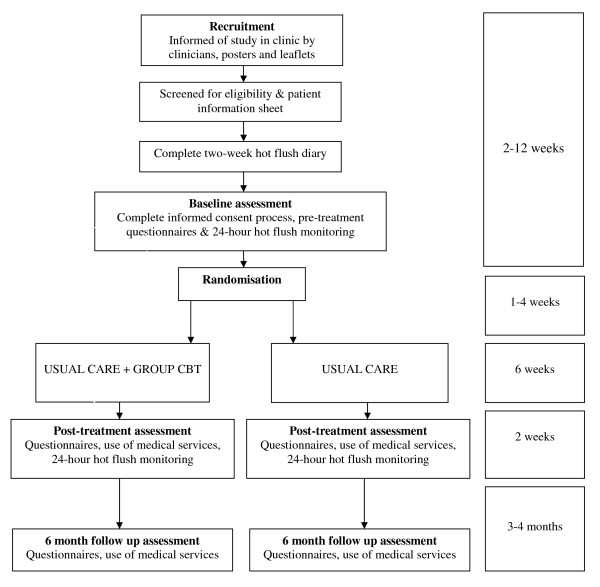
**Description of overall trial design**.

The study design is shown in Figure 1. It is a randomised controlled trial of Group CBT for HF/NS in women who have completed treatment for breast cancer. Participants are block-randomised to receive either usual care (access to clinic support) or to usual care plus six weekly sessions of Group CBT (herein referred to as Group CBT). The Group CBT was developed from previous work and an exploratory trial [45,50] and is specified in a treatment manual. Group CBT is delivered by a clinical psychologist in this trial, but the treatment is designed to be facilitated by appropriately trained and supervised health professionals. Post-treatment assessment is conducted 7-9 weeks post-randomisation (0-2 weeks after 6 weeks of treatment or usual care). Follow up assessment is conducted 6 months post-randomisation. The primary outcome is problem rating of HF/NS at post-treatment assessment. The trial is conducted in compliance with the Helsinki Declaration [51] and is badged with the National Cancer Research Network (UKCRN; Study ID: 4975). NHS REC approval has been granted (South East London 2 REC, ref: 08/H0802/106) and local ethics and R&D approval has been obtained for recruitment of breast cancer patients from all hospitals in the South East London Cancer Research Network (SELCRN). The trial is registered with Current Controlled Trials (ISRCTN13771934). A trial steering committee (TSC) has agreed the research protocol and monitors implementation. Data and participant welfare are monitored by a data management committee (DMC).

### Study sample and recruitment

Participants are recruited from six hospital sites in South East London over an 18 month period from March 2009 to September 2010. Breast cancer patients who report HF/NS at clinic appointments are being recruited into the study by referrals from oncologists, surgeons, radiographers, breast care nurses and SELCRN research nurses working in clinics. Participants are also able to opt in to the study by responding to posters, leaflets and websites. *Inclusion criteria*

1. English speaking patients, aged 18 plus, who have had breast cancer or ducal carcinoma in situ.

2. No evidence of distant metastatic disease.

3. Have had problematic HF/NS for at least 2 months.

4. Have completed active treatment (surgery, radiotherapy and/or chemotherapy) and being in remission, but may be on endocrine treatment such as tamoxifen.

5. Prior medical treatment for HF/NS is recorded. If women have derived partial benefit and are stable on a treatment, they can be included if they have been on this treatment for 2 months or longer.

Women are excluded if depression or general cancer concerns are the main problem that they are seeking help for, not HF/NS, or if they are unable to commit time to attend the sessions.

### Procedure

Women referred from the clinics are contacted by telephone for a screening interview. A researcher conducts an eligibility check using the above criteria and explains what happens in the two arms of the trial and the randomisation process. Those eligible and interested in participating are sent a participant information sheet, a socio-demographic questionnaire and a two-week HF/NS diary to complete, and an appointment for the baseline assessment is arranged. The consent process is completed whereby the researcher summarises the information in the participant information sheet and responds to questions about the study. Participants then provide written consent. Diaries and demographic questions are not collected from those who do not consent or who withdraw consent during baseline assessment. The researcher then conducts a clinical interview covering breast cancer treatment history, medical history including mental health issues, menopause symptoms and treatments used, concomitant medication and therapy use, and current life concerns. Participants are given a questionnaire to complete, assessing HF/NS frequency and problem rating, beliefs about HF/NS, health related quality of life, stress, dispositional optimism and somatisation. After checking that the participant has no allergy to adhesive dressings (e.g. plasters) and does not wear a pacemaker, the SSC hot flush monitor is fitted. Participants are given a magnet event marker, worn on a bracelet, and a 24-hour hot flush diary. They mark the experience of a HF/NS by passing the magnet over the monitor and noting the time and severity of the HF/NS in the diary. Participants wear the monitor for 24 hours, carrying out their usual daily activities and removing the monitor only to shower or bathe.

#### Randomisation

Women are randomised in blocks of 12-20, when sufficient participants have been assessed at baseline. Participant IDs are sent for randomisation by an independent statistician at the Mental Health and Neurology Clinical Trials Unit at King's College London. Women are stratified by age (<50, > = 50) and individually randomised to the two treatment arms in the ratio 1:1, using an internet-based system. Group allocation is sent to the clinical psychologist who delivers the treatment (and therefore cannot be blind to condition). The trial coordinator and trial statistician are not informed of group allocation.

Within four weeks after randomisation, women in the group treatment arm attend six 1 1/2 hour sessions of group CBT, once a week for six weeks. Women from both arms attend post-treatment assessments in the two weeks following the treatment phase (see Figure 1). Post-treatment assessments are conducted by the trial coordinator, who is blind to group allocation. Participants are interviewed about their use of health services and treatments, as well as any changes to lifestyle and health during the treatment phase. They complete post-treatment questionnaires measuring HF/NS frequency and severity, HF/NS beliefs, health related quality of life, stress and somatisation, and repeat the 24-hour SSC hot flush monitoring. Six months after randomisation, participants complete a follow-up questionnaire identical to the post-treatment questionnaire, including use of health services and treatments, and changes in lifestyle and health over the previous four months since post-treatment assessment.

#### Group CBT Intervention

Group CBT comprises 6 weekly sessions of 1.5 hours each. The approach is interactive and psycho-educational, with a focus on helping participants to develop individual treatment strategies through active cognitive and behavioural change. Each session is run according to protocol set out in a treatment manual, including presentation slides and handouts. A clinical psychologist has been trained to deliver the treatment and assistants also are trained to assist in the groups. Sessions are taped and 2 independent psychologists, experienced in cognitive behavioural therapy, rate 10% of the sessions for adherence to protocol using a coding sheet created for the purpose. The components of each session are listed in Table 1. The change techniques used in each component are categorised using a 40-item revised version of Abraham and Michie's 26-item taxonomy of behaviour change techniques [52,53]. At each session participants create an action plan of homework to carry out between sessions. At the start of each session participants feed back their experiences of implementing changes during the previous week. Where difficulties are encountered, the group is encouraged to provide support and help the participant in problem-solving. Positive effects of changes are highlighted and discussed. Participants continue to practice, monitor and implement changes from previous weeks in addition to the new action plan each week. Paced breathing and relaxation are practiced within each session and as homework using a CD (developed for the treatment).

**Table 1:** Intervention techniques used in the Group CBT treatment

Intervention Component	Change Technique
SESSION 1	
▪ Participants discuss experiences of breast cancer, HF/NS, and personal goals for treatment	28, 6
▪ Provide information about physiology of HF/NS and the role of thoughts, feelings and behaviours	1
▪ Discuss triggers of HF/NS and ways to modify them	28, 8
▪ Introduction to full body relaxation	21, 26
HOMEWORK:	
▪ Monitor and modify triggers of HF/NS	16, 17
▪ Practice full body relaxation	9, 26

SESSION 2	
▪ Participants feedback on homework from previous session, with group support and problem solving of difficulties and focus on positives	19, 28, 29, 8, 18
▪ Provide information on stress and discuss its impacts on HF/NS	36, 1, 2
▪ Provide examples of cognitive and behavioural stress management techniques	36, 21
▪ Participants create a specific stress goal to enhance wellbeing	7
▪ Introduce paced breathing to relaxation	21, 26
HOMEWORK as before plus:	
▪ Implement stress goal	7
▪ Practice paced breathing	9, 26

SESSION 3	
▪ Participants feed back on homework from previous sessions, as above	19, 28, 29, 8, 18
▪ Discuss cognitive, emotional and behavioural reactions to HF/NS and generate more helpful responses	8
▪ Participants try out some cognitive and behavioural strategies for managing HF/NS	26
▪ Introduce use of paced breathing and cognitive strategies at onset of HF/NS to reduce impact of flush	21, 26
HOMEWORK as before plus:	
▪ Monitor thoughts, feelings and behavioural reactions to HF/NS, practice more helpful reactions	17, 26
▪ Practice paced breathing at the onset of a HF/NS	9, 26
▪ Complete a sleep diary for a week	17

SESSION 4	
▪ Participants feedback on homework from previous sessions as above	19, 28, 29, 8, 18
▪ Provide information on sleep and discuss sleep diaries	1, 2
▪ Discuss behavioural strategies for improving sleep quality	21
▪ Participants create an action plan to implement two sleep goals	7
▪ Practice relaxation and paced breathing	21, 26
HOMEWORK as before plus:	
▪ Implement sleep goals	24

SESSION 5	
▪ Participants feedback on homework from previous session as above	19, 28, 29, 8, 18
▪ Provide information on thoughts and sleep	1
▪ Provide tips on managing cognitive and emotional reactions to sleep difficulties	21
▪ Create action plan to change thinking and behaviour when having sleep difficulties	7
▪ Practice Relaxation and Paced breathing	21, 26
HOMEWORK as before plus:	
▪ Implement second set of sleep goals	7

SESSION 6	
▪ Participants feedback on homework from previous sessions as above	19, 28, 29, 8, 18
▪ Discuss overall progress and write a therapy blueprint by identifying helpful strategies, potential barriers or setbacks, ways of spotting setbacks and overcoming them, and a maintenance plan for future goals relating to the treatment	35, 16, 17
▪ Group discussion of managing uncertainty and future plans, and symptoms in the context of breast cancer	29, 28
▪ Practice Relaxation and Paced breathing	21, 26
▪ Goodbye and how to access help in the future	29

Notes:

1: Provide information on consequences of behaviour in general*; 2: Provide information on the consequences of behaviour to the individual*; 6: Goal setting (outcome); 7: Action planning; 8: Barrier identification/Problem solving; 9: Set graded tasks; 16: Prompt self-monitoring of behaviour; 17: Prompt self-monitoring of outcome; 18: Prompting focus of past success; 19: Provide feedback on performance**; 21: Provide instruction on how to perform a behaviour; 24: Environmental restructuring; 26: Prompt practice; 28: Facilitate social comparison; 29; Plan social support; 35: Relapse prevention/Coping planning; 36: Stress Management.

*The definition is interpreted broadly to include consequences of cognition and emotion. According to cognitive behaviour theory there are cognitive and emotional consequences and antecedents of behaviour which impact upon health outcomes (in this case HF/NS).

**The term performance may be slightly misleading as it suggests that feedback should be judgemental (good or bad), although it is not specifically defined this way in the taxonomy.

The definitions of change techniques are available on request from Stephanie Ashford (s.ashford@coventry.ac.uk).

*Session 1 *aims to engage participants by introducing them to the CBT model of HF/NS [16,46]. Participants discuss their experiences of menopause and personal goals for the treatment. They are informed about, and discuss, physiological, cognitive, behavioural, and emotional components of HF/NS. This is embedded within an overall CBT model of menopausal symptoms that acknowledges the effect of stress upon the hot flush threshold. Common triggers are discussed and for homework participants monitor HF/NS for triggers and experiment by modifying them. Full body relaxation is introduced in the first session without paced breathing, and participants practice relaxation for homework.

*Session 2 *reviews homework and introduces a cognitive behavioural model of stress and stress management using a series of interactive exercises and discussion. Cognitive therapy is used to increase awareness of the role of unhelpful and overly negative thoughts and to facilitate calm responses. Behavioural strategies are encouraged to promote well-being and to reduce stress. Participants are asked to identify a stress reducing goal and implement it as homework. Paced breathing (slow, deep and even breathing with attentional focus on the breathing) is introduced to the relaxation session and participants are prompted to continue practice of paced breathing and relaxation as homework.

*Session 3 *focuses on cognitive and behavioural aspects of hot flushes. Participants revisit the CBT model introduced in session 1 and information about psychological processes contributing to distress during a hot flush are introduced. Cognitive behavioural strategies for managing hot flushes during social situations, and in relation to a perceived loss of control are discussed. Participants create an action plan to implement a cognitive behavioural strategy as homework. The role of paced breathing in reducing the impact of a hot flush is introduced and participants are asked to practice paced breathing at the onset of a flush for homework. This is suggested within an approach of accepting the flush (not fighting it), and letting the flush flow over them as they breathe; in doing so the focus of attention is on the body and breathing rather than thoughts. They then redirect attention to their activities.

*Session 4 *is the first of two sleep sessions, and lays the foundations for managing night sweats by improving general sleep quality. Participants are taught the mechanisms of sleep and cognitive processes that influence subjective judgements of sleep to address anxieties about sleep. Behavioural strategies (adapted from Harvey [54]) are introduced and participants are asked to create a sleep goal (action plan) and implement it as homework.

*Session 5 *builds on behavioural sleep strategies by adding in the cognitive component. Participants discuss cognitive strategies to address sleep-related and general anxieties leading to wakefulness. Calm management of night sweats is encouraged that incorporates paced breathing. Strategies to deal with overly anxious thinking are discussed. Participants are asked to identify and implement a relevant sleep goal as homework.

*Session 6 *reviews the overall model and goals of the previous five weeks. Participants write and share individual maintenance plans by considering cognitive and behavioural changes made through psycho-education and homework tasks. They make action plans to maintain changes and identify potential barriers or setbacks and ways of overcoming them. This session also schedules some time for discussion of menopause-related topics not covered in previous sessions, chosen by the participants, which may include weight gain, memory, adjusting to life after breast cancer, and sexuality.

#### Usual care

The control arm receives standard care, that is, they have access to their oncologist and clinical nurse specialist, as well as cancer information and support services. Treatments and services accessed during the treatment phase are logged at post-treatment assessment. The usual care group are offered a form of CBT off-trial at the end of the trial.

### Measures

The measures taken at each stage of the trial are summarised in table 2.

**Table 2:** Study measures

Phase	Pre-baseline	A0 Baseline assessment	Treatment phase	A1 Post-treatment assessment	A2 Follow up assessment
**Interview measures**					
Medical history incl. breast cancer treatment, menopause symptoms, concomitant medications and therapies		X			
**Questionnaire measures**					
Weekly diaries	X^1^		X^2^		
Demographics: Age, BMI, ethnicity, parity, marital status, education, employment, smoking, drinking, exercise		X			
Hot flush rating scale		X	X^2^	X	X
Hot flush beliefs scale		X		X	X
Women's health questionnaire		X		X	X
Perceived Stress Scale		X		X	X
Somatic Amplification Scale		X		X	X
SF-36		X		X	X
Life Orientation Test		X			
Use of services				X	X
CBT session evaluation				X^3^	
**24 hour monitoring**					
Sternal skin conductance		X		X	
24 hour diary		X		X	

Notes:

^1^Two weekly diaries completed prior to baseline assessment

^2^Completed each week for 6 weeks by those randomised to Group CBT only

^3^Completed by participants receiving Group CBT only

#### Socio-demographics

Socio-demographic variables include date of birth, height, weight, ethnicity, number of children, education, marital status, employment status, smoking, drinking and exercise. Breast cancer treatment, use of treatments for menopause and concomitant medications and therapies are also recorded.

#### Primary Outcome: Hot flush problem rating

Hot flush problem rating is a subscale of the Hot Flush Rating Scale [55], calculated as the mean of three items each measured on a 10 point scale (low to high), e.g. "To what extent do you regard your flushes/sweats as a problem?" (1 = not at all a problem, 10 = very much a problem).

#### Secondary Outcomes

Hot flush frequency and severity are assessed using the following measures:

##### Hot Flush Rating Scale [55]: Frequency subscale

The frequency subscale of the HFRS is a retrospective estimation of the number of HF/NS experienced in the previous week, e.g. "How often have you had hot flushes in the past week?" Average severity of HF/NS over the previous week is estimated for hot flushes and for night sweats separately (1 = mild, 2 = moderate and 3 = severe).

##### 24-hour hot flush diary

Women monitor their HF/NS prospectively for 24 hours. When they experience a hot flush or night sweat they note down the severity (1 = mild, 2 = moderate and 3 = severe) and the time at which it occurred.

##### 24-hour ambulatory sternal skin conductance and event marker

During the 24-hour diary monitoring period women wear an ambulatory SSC monitor [56]. The monitor is approximately 6 × 6 cm, with two 8 cm electrodes attached to an adhesive electrode patch by press studs. The adhesive electrode patch is attached to the sternum, two inches below the sternal notch, which holds the monitor close to the body. Skin conductance is measured by passing a pulse through the electrodes every 5 seconds, indicated by a green flash of an LED on the monitor. A hot flush is defined by an increase in conductance of 2 mmhos or more in 30 seconds, occurring no less than 15 minutes since the last SSC-defined HF/NS (i.e. 15 minute lock out period [57]). For each SSC-defined hot flush or night sweat, SSC level and the time at which it occurred are extracted for analysis. Women can indicate that they are experiencing a HF/NS by passing a magnet near to the monitor. The magnet is worn on a bracelet. The times at which the magnetic event marker is registered are summed to produce a 24 hour HF/NS frequency.

##### Health related quality of life

General physical and mental health are indexed using the *General Health Survey Short Form 36 *(SF-36) [58], a widely used 36 item health survey. Two physical and mental health summary scales are constructed, with a possible range from 0 to 100, where higher scores indicate better health [59]. Perceptions of physical and emotional symptoms are measured using the *Women's Health Questionnaire *(WHQ) [49] which is standardised on samples of mid-aged women. The WHQ contains 36 items, for example, "I feel more tired than usual", which are scored on 4-point scales. Nine subscales are formed by counting the number of items scoring 2 or 3 and dividing by the number of items for each scale, resulting in scales from 0 to 1. The WHQ has been widely used to evaluate interventions for menopausal symptoms [60].

#### Process measures

##### Beliefs about hot flushes and night sweats

HF/NS beliefs are measured using the Hot Flush Beliefs Scale [15], a 27-item scale comprising three subscales including social context, coping with hot flushes, and coping with night sweats. An additional scale is included which measures behavioural reactions to HF/NS, the Hot Flush Behaviour Scale (HFBehS).

##### Perceived stress

The Perceived Stress Scale [61] measures 10 items, for example "How often have you been upset because of something that happened unexpectedly?", on a scale from 0 (never) to 4 (very often). Items are summed to form a 0-40 scale, where 0 is low stress and 40 is high stress.

##### Somatic amplification

Barsky, Grace and Klerman's [62] Somatic Amplification Scale is a 10 item scale measuring a general tendency toward body focus, including items such as "When someone else coughs, it makes me cough too" (1 = not like me to 5 = very much like me). The mean of the 10 items is calculated resulting in a 5-point scale where 5 is high somatic amplification.

##### Dispositional optimism

The Revised Life Orientation Test (LOT R [63]) measures dispositional optimism on a 8-item scale including "In uncertain times, I usually expect the best" (0 = strongly disagree to 4 = strongly agree). A mean of all items is calculated, resulting in a 5-point scale where high scores indicate higher dispositional optimism.

##### Treatment evaluation

Participants randomised to the group CBT arm are asked to rate the usefulness of the sessions overall and of specific elements of the intervention; use of the relaxation CD, attendance and completion of homework are also being measured.

### Adverse events

An adverse events protocol has been agreed by the DMC and TSC, adapted for a non-medical trial using the Medical Research Council/Department for Health Joint Project Guideline notes on Pharmocovigilance [64]. This excludes study outcomes, such as increases in frequency or severity of HF/NS that occur as part of normal fluctuations in menopause symptoms. Adverse events are logged in the trial master file and reported to the principle investigator and trial coordinator. In accordance with the National Patient Safety Agency's requirements for reporting of serious adverse events (SAE), any adverse event deemed to be a related or unexpected SAE will be reported to the principle investigator, the sponsor and the DMC within 48 hours, and reported to the Hospital Research Ethics Committee in accordance with their procedures. Reoccurrence of breast cancer or secondary cancer does not need to be reported in this way, as it is an expected unrelated serious adverse event (see appendix 3 of the Joint Guidelines [64]). An adverse events report of these occurrences will be included in the report to the DMC in accordance with the DMC charter.

### Power calculation

The sample size is calculated on the HFRS problem rating subscale [55], estimating a mean of 5 (SD = 2.4) and a clinically relevant difference of 2 points [45] at two weeks post-treatment. A total sample size of 76, 38 in each group, will have 90% power to detect a difference in mean HF/NS Problem Rating of 2, controlling for baseline value, assuming a common standard deviation of 2.4, a correlation between baseline and end of treatment values of 0.4 (giving a correction factor of 0.84 [65]) and a 5% two-sided significance level. Due to clustering of outcomes within therapy groups, the sample size has been adjusted by assuming an intra-class correlation of 0.07 [66] and 8 participants per group giving a variance inflation factor of 1.49. Ninety six women will be recruited in to the trial to allow for a 20% attrition rate.

### Analysis

An analysis plan has been agreed (i.e. prior to completion of data collection). A trial statistician, employed by the Mental Health and Neurology Clinical Trials Unit at King's College London, carries out primary and secondary data analysis, and is blind to group allocation. Unless indicated, scales are calculated according to published scoring algorithms. All statistical tests are two sided (level of significance = 0.05). The analyses are preformed using the statistical software Stata version 11.0. An intention to treat analysis of the primary outcome is performed. Difference in problem rating between the two intervention groups is tested using a linear mixed model, utilising fixed and random effects. The regression model compares the problem rating subscale of HFRS between intervention groups, after allowing for the baseline measurements to control for pre-treatment differences, stratification factors (age: 50 years or older versus younger than 50 years), and clinically relevant covariates. Greater precision of estimates is expected within therapy groups. Therefore models are adjusted for the therapy group; this variable is able to randomly vary around the intercept (random effects). Secondary outcomes at post-treatment are analysed similarly. Follow-up data at 6 months is also explored through linear mixed models utilising repeated measures analyses, allowing simultaneous modelling of the two outcome time points. Mediation of treatment effects by hot flush beliefs and behaviours, mood, stress, quality of life and frequency of HF/NS defined by skin conductance rises will be explored using a two stage least squared method in Stata [67]. Moderation by optimism and somatisation is also tested using multiple-group modelling [68].

## Discussion

This study is a randomised controlled trial of cognitive behavioural therapy to treat hot flushes and/or night sweats experienced by women who have completed treatment for breast cancer. This non-medical treatment aims to alleviate HF/NS symptoms in women who have limited medical treatment options due to contraindications. It is a robustly designed trial, comparing six sessions of Group CBT to usual care using both self-reported and physiological outcome measures. The primary outcome is change in HF/NS problem rating at post-treatment: an average reduction in problem rating of 2 points on the HFRS problem rating subscale is needed for treatment efficacy to be considered clinically significant. The study also tests the hypotheses that Group CBT will reduce frequency of HF/NS, improve mood, sleep, general wellbeing, and beliefs about HF/NS. It is further hypothesised that these improvements will be maintained at six months post-randomisation. If this non-medical treatment is found to be successful it could be delivered in primary or secondary care settings by appropriately trained and supervised health professionals.

### Methodological considerations

The design has several strengths. The treatment has been extensively developed and piloted for feasibility, acceptability and preliminary outcomes. Preliminary results found an average 2.5 point reduction in problem rating (49% reduction) as well as a significant reduction in self-reported frequency, and significant improvements in sleep quality and quality of life were also reported [47]. A treatment manual specifies the detail for each session so that it is replicable, and checks are in place to ensure adherence to protocol. Measures of treatment efficacy are robust: as well as questionnaire-based estimations of recent symptoms, the trial includes prospective monitoring of symptoms using self-report and physiological measures. Very few trials of treatments for HF/NS include both types of prospective monitoring, and no trials of CBT for HF/NS have done so. The study sample is drawn from a socially and culturally diverse area of the UK; the uptake and adherence to the treatment by women from different backgrounds will be reported.

There are some limitations that should be considered. Recruitment procedures are designed to reach all of the symptomatic breast cancer patients who attend clinics in the South East London Network (SELCRN) hospitals. Clinic and research staff approach patients directly and posters and leaflets are placed in waiting rooms so that patients can opt in themselves. Nonetheless, the sample might not be representative of symptomatic breast cancer patients in general and we cannot know how many people were made aware of the trial through these recruitment procedures.

The treatment is compared to a usual care group to see whether it improves on current practice, but usual care control groups have some drawbacks. Usual care may vary between centres, although typically treatment is minimal. Those receiving usual care may be more likely to seek complimentary therapies if they are having no other treatment. Complementary therapy use and clinic treatments are monitored in the trial and may have to be controlled for in analysis. If usual care is minimal then it will be similar to a no-treatment control group, and treatment effects might be due to the extra attention rather than the treatment per se (i.e. any intervention is better than nothing). Transitory changes and biased reporting should be apparent by comparing self-reported HF/NS with physiological measures and post-treatment with follow up data. Early in the treatment development phase it makes sense to compare with current practice, especially as some improvement in usual care would be predicted if only because symptoms do get better in time. Should treatment effects be robust, further research into the active treatment components can be tested using attention control groups.

## Conclusions

Breast cancer survivors who are experiencing problematic HF/NS have few treatment options. Cognitive behavioural therapy has resulted in some promising reductions in HF/NS problem rating and frequency in these women. This study is a randomised controlled trial of Group CBT for HF/NS which also includes physiological outcome measures. The trial tests for treatment effects on HF/NS, sleep, mood, beliefs about HF/NS and general wellbeing. This study represents an important step in the development of a cost-effective non-medical treatment for HF/NS designed to be implemented on a wider scale by trained and supervised health professions.

## Abbreviations

CBT: Cognitive behavioural therapy; HF/NS: hot flushes and/or night sweats; HFRS: Hot flush rating scale; HFBS: Hot flush beliefs scale; SF-36: General Health Survey Short form 36; WHQ: Women's Health Questionnaire; LOT R: Revised Life Orientation Test; TSC: Trial Steering Committee; DMC: Data Management Committee; NHS: National Health Service; UKCRN: United Kingdom Cancer Research Network; SELCRN: South East London Cancer Research Network; RCT: Randomised controlled trial; SSC: Sternal skin conductance; HT: Hormone therapy (previously termed hormone replacement therapy or HRT).

## Competing interests

The authors declare that they have no competing interests.

## Authors' contributions

EM is the trial coordinator on the study and generated the first draft of this manuscript based on the study protocol; MS contributed to the intervention manual and carried out the Group CBT intervention; JH is the statistician to the study; MSH is the principal investigator of this study and wrote the original protocol and supervised the intervention. All authors approved the final version of the manuscript.

## Pre-publication history

The pre-publication history for this paper can be accessed here:

http://www.biomedcentral.com/1471-2407/11/44/prepub
